# Gender dimorphism of white matter integrity assessed by diffusion tensor magnetic resonance imaging in abstinent alcoholic men and women

**DOI:** 10.1186/1940-0640-10-S1-A59

**Published:** 2015-02-20

**Authors:** Kayle S Sawyer, George Papadimitriou, Susan M Ruiz, Nikos Makris, Marlene Oscar-Berman, Gordon J Harris

**Affiliations:** 1 Department of Anatomy & Neurobiology, Boston University School of Medicine, Boston, MA, 02118, USA; 2 Psychology Research Service, VA Boston Healthcare System, Jamaica Plain, MA, 02130; 3 Center for Morphometric Analysis, Massachusetts General Hospital, Charlestown, MA, 02129, USA; 4 Departments of Psychiatry and Neurology, Boston University School of Medicine, Boston, MA, 02118, USA; 5 3D Imaging Service, Massachusetts General Hospital, Boston, MA, 02114, USA

## Background

Alcoholism is a debilitating disorder associated with widespread cognitive and neurological abnormalities. However, there is limited scientific literature evaluating gender-specific similarities and differences in microstructural white matter pathology associated with alcoholism. In our prior work, we used diffusion tensor magnetic resonance imaging to examine the integrity of white matter fiber tracts in the brains of abstinent alcoholic (ALC) men compared with nonalcoholic (NC) men. We found that ALC men had decreased fractional anisotropy (FA) within white matter fiber tracts connecting to frontal and limbic networks, primarily of the right hemisphere (Harris et al., 2008). In the current project, we sought to confirm our prior findings in abstinent ALC men and, additionally, examine whether different white matter abnormalities were present in abstinent ALC women.

## Methods

As determined by our manual inspection, 60-direction high-quality diffusion tensor imaging images were acquired from 30 abstinent (at least 4 weeks) ALC participants (21 women) and 25 NC controls (17 women). Tract-based spatial statistics tools included in FSL 5.0 were used to analyze a tensor model that yielded regional FA values for each participant. To examine the effects of gender, we built a 2 X 2 ANOVA design with three planned comparisons of primary interest: ALC group-by-gender interaction, ALC women versus NC women, and ALC men versus NC men.

## Results

We observed FA deficits in ALC men relative to NC men, with a similar effect size and variability as observed in our prior study. In contrast, ALC women displayed strikingly greater FA values compared to NC women in widespread white matter regions, including most principal long-association fiber tracts. Also, they had greater FA for local white matter architecture in the dorsolateral and ventral prefrontal regions, as well as the sublenticular extended amygdala. When controlling for multiple comparisons, the higher FA observed in ALC women remained significant. For many regions, group-by-gender interaction effects were observed. However, likely due to the small sample sizes for men, the interaction effects did not survive threshold-free cluster enhancement, the correction procedure for multiple comparisons used in these analyses.

## Conclusions

These results suggest antithetical gender abnormalities in white matter tracts of ALC brains. Whereas abstinent ALC men displayed deficits consistent with our prior study, our new findings for abstinent ALC women demonstrated increased FA values. These distinct patterns of white matter abnormalities point toward a differential underlying neural basis for gender-specific propensity and/or sequelae to long-term alcoholism, and suggest implications for further investigation of possible gender-specific approaches to prevention and treatment.
